# An Audit of Antidepressant Prescribing in a Single-Centre Child and Adolescent Mental Health Service (CAMHS)

**DOI:** 10.1192/bjo.2024.616

**Published:** 2024-08-01

**Authors:** Cara Pattiata, Anthea Prosser

**Affiliations:** Tees, Esk and Wear Valley NHS Foundation Trust, Darlington, United Kingdom

## Abstract

**Aims:**

The audit aims to check compliance of prescribers to the following National Institute of Clinical Excellence (NICE) guidelines:

- NG134, CG31: Antidepressants are prescribed in conjunction with psychological therapy.
- NG134: A risk-benefit discussion took place.
- NG134 Written information was given.
- NG134, CG31: First-line medication was prescribed in the first instance.
- NG134: An off-licence medication is only prescribed after a review.
- NG134: A consent form is signed if an off-licence is prescribed.

**Methods:**

All patients under CAMHS and receiving antidepressant therapy was considered. People on the caseload currently an inpatient were excluded. The audit was performed in October 2023. 86 eligible patients were randomised; 30 were selected for case review. Clinic letters and internal case notes were reviewed to check compliance.

**Results:**

Areas of good compliance: antidepressants prescribed with psychological therapy, risk-benefit discussions took place, first-line medications prescribed in the first instance, off-licence medications prescribed only afer review.

Areas of moderate compliance: written information given with prescriptions.

Areas of no compliance: consent form does not form part of standard practice or local guidelines.

**Conclusion:**

The local CAMHS service showed good compliance to NICE guidelines around antidepressant prescribing. Presentation to the local team is required to remind clinicians of the need to document parts of the consultation such as giving written information. A discussion with the regional consultant body yielded the outcome that the service will adhere to local Trust guidelines of internal case notes documenting consent rather than a signed form. The standards for the re-audit in 6 months will reflect this.

